# Comparison of the WHO Labour Care Guide Versus a Modified Partograph for Maternal and Foetal Outcomes at a Rural Care Centre: A Preventive Controlled Trial

**DOI:** 10.7759/cureus.105214

**Published:** 2026-03-14

**Authors:** Pragati Divedi, Pallavi Tripathi, Kalpana Kumari, Soniya Vishwakarma, Nupur Mittal, Sarika Pandey

**Affiliations:** 1 Obstetrics and Gynecology, Santosh Medical College and Hospital, Ghaziabad, IND; 2 Obstetrics and Gynecology, Uttar Pradesh University of Medical Sciences, Etawah, IND

**Keywords:** fetal outcome, labour management, maternal outcome, modified partograph, who labour care guide

## Abstract

Objective

This study aimed to compare the WHO Labour Care Guide (WLCG) with the Modified Partograph concerning maternal and foetal outcomes in a rural healthcare setting.

Methods

A prospective comparative study was conducted on 100 labouring women admitted to a rural maternity centre. Of these, 50 women were managed using the WLCG, while the other 50 were managed using the Modified Partograph. Data on demographic characteristics, labour progression, maternal outcomes, and foetal outcomes were collected and statistically analysed.

Results

The demographic characteristics, including age (p = 0.586), parity (p = 0.294), socioeconomic status (p = 0.158), and mode of labour onset (p = 0.680), showed no significant differences between the two groups. The duration of the second stage of labour was significantly shorter in the WLCG group (14.83 ± 7.78 minutes) compared to the Modified Partograph group (21.73 ± 10.53 minutes, p = 0.001). No significant difference was noted in the duration of the active stage (p = 0.075) or mode of delivery (p = 0.295). A significantly higher percentage of women reported a better birth experience in the WLCG group (p < 0.001). The causes of intervention (p = 0.743), APGAR scores (p = 0.907), neonatal mortality (p = 0.603), and maternal complications (p = 0.419) were comparable. However, NICU admissions were significantly lower in the WLCG group (30% vs. 52%, p = 0.025).

Conclusion

The WLCG demonstrated advantages in reducing the second stage of labour duration, improving women's birth experiences, and lowering NICU admission rates compared to the Modified Partograph, without compromising maternal and foetal safety. This suggests the potential benefits of adopting WLCG in rural obstetric care settings.

## Introduction

Efficient labour monitoring is crucial for optimising maternal and foetal outcomes. Traditionally, the Modified Partograph has been the standard tool used in labour wards to monitor labour progress and identify complications. The WHO Labour Care Guide (WLCG) was developed as a more dynamic and evidence-based tool intended to improve intrapartum care and maternal satisfaction. However, comparative studies evaluating these two tools, especially in rural settings, remain limited. This study aims to compare the maternal and foetal outcomes using the WLCG and the Modified Partograph among women delivering in a rural healthcare facility. It is essential that the LCG incorporates appropriate parameters for labour monitoring and is capable of addressing the practical needs of maternity care providers across various healthcare settings [1]. These WHO recommendations include new definitions and durations of the first and second stages of labour and highlight the importance of woman-centred care to optimise the experience of labour and childbirth for women and their babies [2].

The partograph has to be revised to accommodate treatment in accordance with new research and international priorities for intrapartum care for a positive childbirth experience [3]. It is essential that the LCG incorporates appropriate parameters for labour monitoring and is capable of addressing the practical needs of maternity care providers across various healthcare settings [4]. Both the partograph and the WLCG emphasise the structured and regular documentation of key clinical parameters that reflect the health status of both the mother and the foetus during labour [5].

## Materials and methods

Study design, population, and sample size

It is a hospital-based preventive controlled study. All antenatal women in the first stage of labour with low obstetric risk admitted to the emergency labour room of the Department of Obstetrics and Gynaecology were included in the study. A total of 100 participants were recruited and divided into two groups: 50 managed using the WLCG and 50 using the Modified Partograph.

Methodology

**Figure 1:**
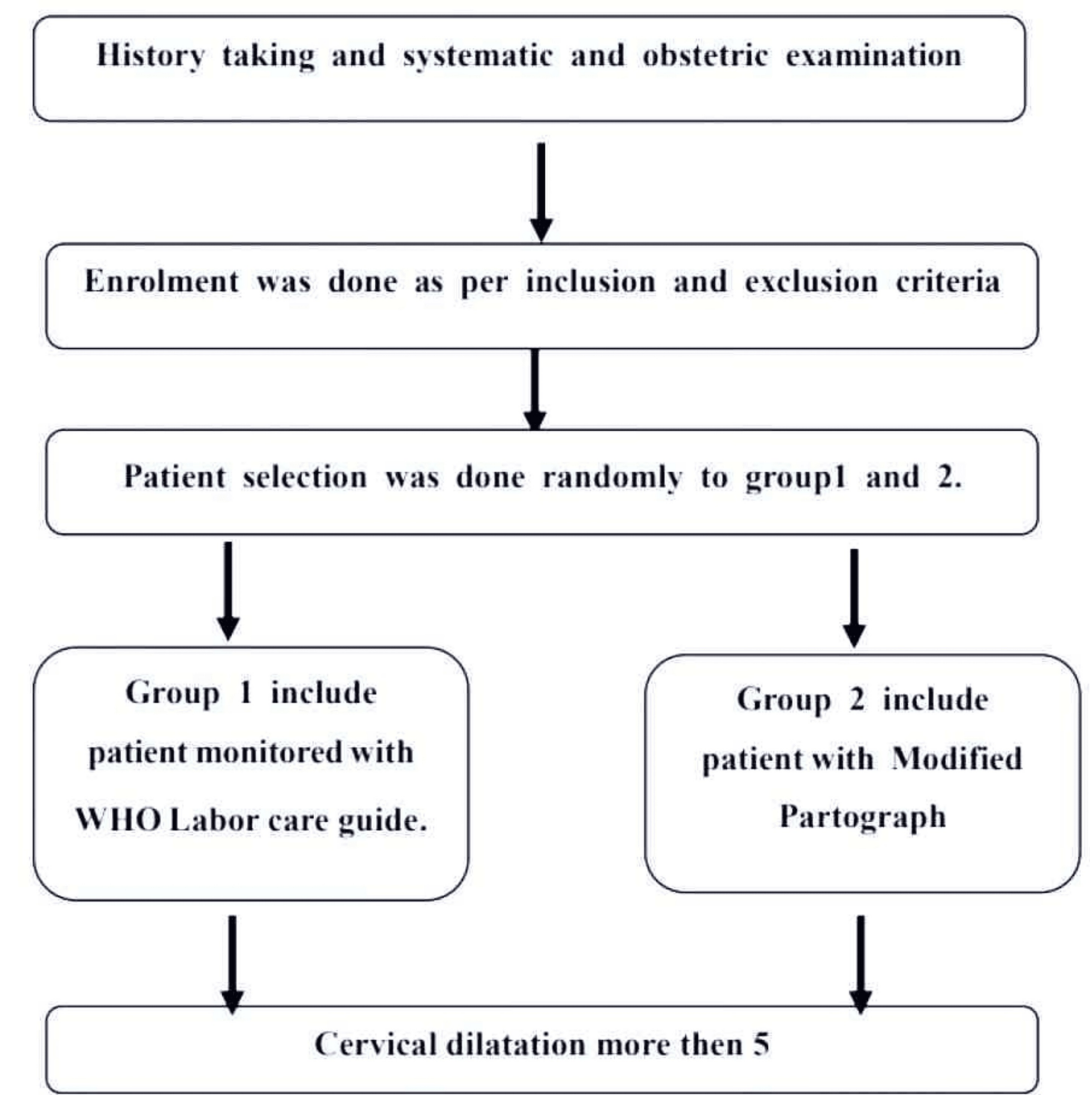
Methodology

History taking, systematic, and obstetric examinations were done. Enrolment was done as per the inclusion and exclusion criteria. Patient selection was done randomly into Groups 1 and 2. Group 1 included patients monitored with the WLCG. Group 2 included patients monitored with the Modified Partograph. Both groups included patients with cervical dilatation of 5 cm (Figure 1).

**Figure 2:**
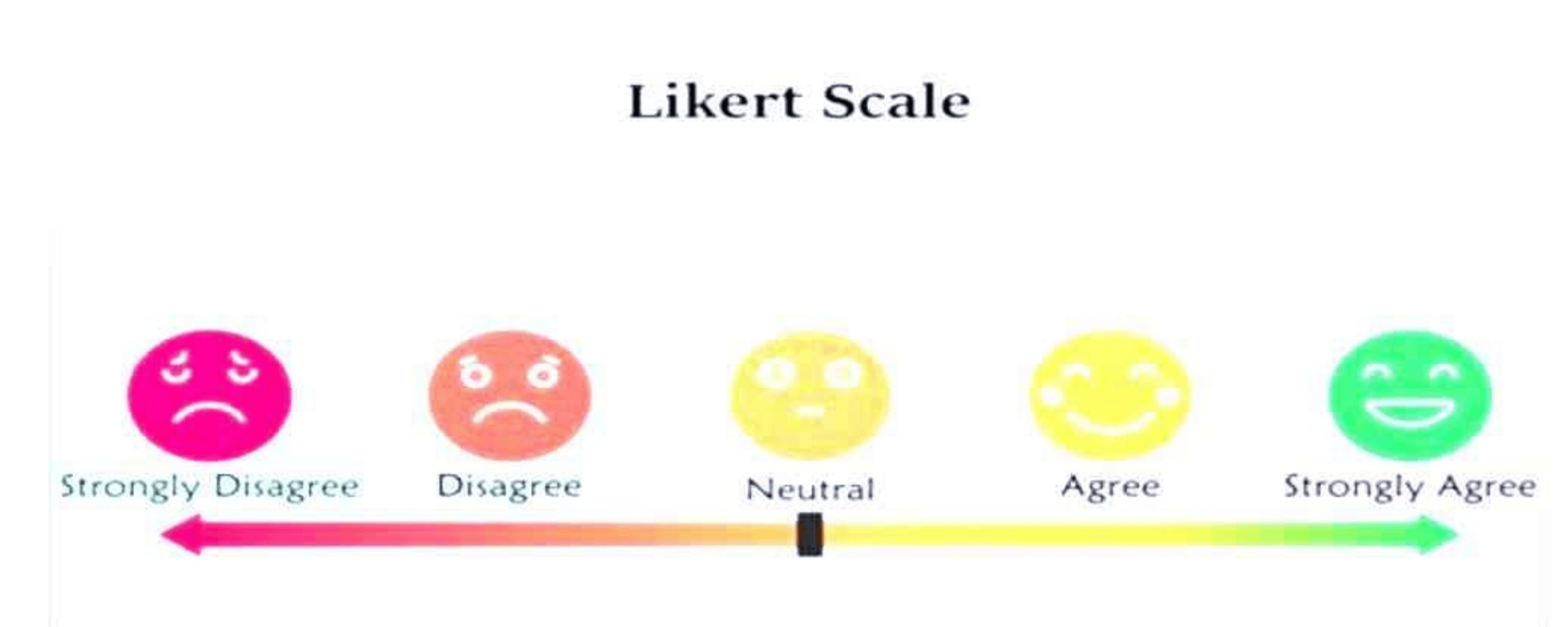
Likert scale

Maternal and neonatal outcomes were followed until 48 hours in postnatal care. Data regarding demographic characteristics, labour progression, maternal outcomes, and foetal outcomes were analysed and compared between the two groups. Women’s experience at birth was analysed using a five-point Likert scale (Figure 2).

Statistical analysis

The data entry was recorded in a Microsoft Excel spreadsheet (Microsoft® Corp., Redmond, WA, USA), and the final analysis was performed using IBM SPSS Statistics for Windows, Version 21 (Released 2012; IBM Corp., Armonk, NY, USA). The Chi-square (χ²) test was used to compare the progress of labour and outcomes between primipara and multipara, and the p-value was calculated to determine the level of significance. A non-parametric statistical test (Wilcoxon signed-rank test) was also used wherever applicable.

Ethics statement and confidentiality

Institutional Ethics Committee approval and written informed consent in the patient’s own language were obtained (approval no. 11\2023-24). Information procured from the study was kept confidential and used for academic purposes only.

## Results

Characteristics, including age, parity, socioeconomic status, and mode of labour onset, were comparable between the groups with no significant differences.

The difference in parity distribution between the two groups was not statistically significant (p = 0.294), indicating comparable parity characteristics (Table 1). The difference in socioeconomic status distribution between the two groups was not statistically significant (p = 0.158), indicating comparable socioeconomic backgrounds (Table 2).

**Table 1:** Parity distribution in each group

Parity	WHO Labour Care Guide, N (%)	Modified Partograph, N (%)	p-value
0	23 (46.0%)	17 (34.0%)	0.294
1	15 (30.0%)	24 (48.0%)	
2	8 (16.0%)	7 (14.0%)	
3	4 (8.0%)	2 (4.0%)	
Total	50 (100%)	50 (100%)	

**Table 2:** Socioeconomic status distribution among each group

Socioeconomic Status	WHO Labour Care Guide, n (%)	Modified Partograph, n (%)	p-value
Lower	22 (44.0%)	25 (50.0%)	0.158
Lower Middle	7 (14.0%)	4 (8.0%)	
Middle Class	15 (30.0%)	8 (16.0%)	
Upper	0 (0.0%)	2 (4.0%)	
Upper Middle	6 (12.0%)	11 (22.0%)	
Total	50 (100%)	50 (100%)	

The second stage of labour was significantly shorter in the WLCG group (14.83 ± 7.78 minutes) compared to the Modified Partograph group (21.73 ± 10.53 minutes, p = 0.001). There was no significant difference in the duration of the active stage of labour (p = 0.075). For the active stage of labour, the mean duration in the WLCG group was 4.80 ± 1.53 hours, while in the Modified Partograph group, it was slightly shorter at 4.32 ± 1.12 hours, with a combined mean of 4.56 ± 1.35 hours. The difference in the active stage duration between the two groups was not statistically significant (p = 0.075), suggesting comparable active stage durations in both groups (Table 3).

**Table 3:** Duration of labour stages in each group

Group	Duration of 2nd Stage of Labour (min), Mean ± SD	Duration of Active Stage of Labour (hr), Mean ± SD
WHO Labour Care Guide	14.83 ± 7.78	4.80 ± 1.53
Modified Partograph	21.73 ± 10.53	4.32 ± 1.12
Total	18.16 ± 9.79	4.56 ± 1.35
p-value	0.001	0.075

The difference in the cause of intervention between the two groups was not statistically significant (p = 0.743), indicating a similar intervention rate and cause distribution across both groups (Table 4).

**Table 4:** Cause of intervention in each group NA, not applied; NPOL, non-progression of labour; DTA, deep transverse arrest

Cause of Intervention	WHO Labour Care Guide, n (%)	Modified Partograph, n (%)	p-value
NA	47 (94.0%)	45 (90.0%)	0.743
Foetal Distress	2 (4.0%)	3 (6.0%)	
NPOL	1 (2.0%)	1 (2.0%)	
DTA	0 (0.0%)	1 (2.0%)	
Total	50 (100%)	50 (100%)	

**Figure 3:**
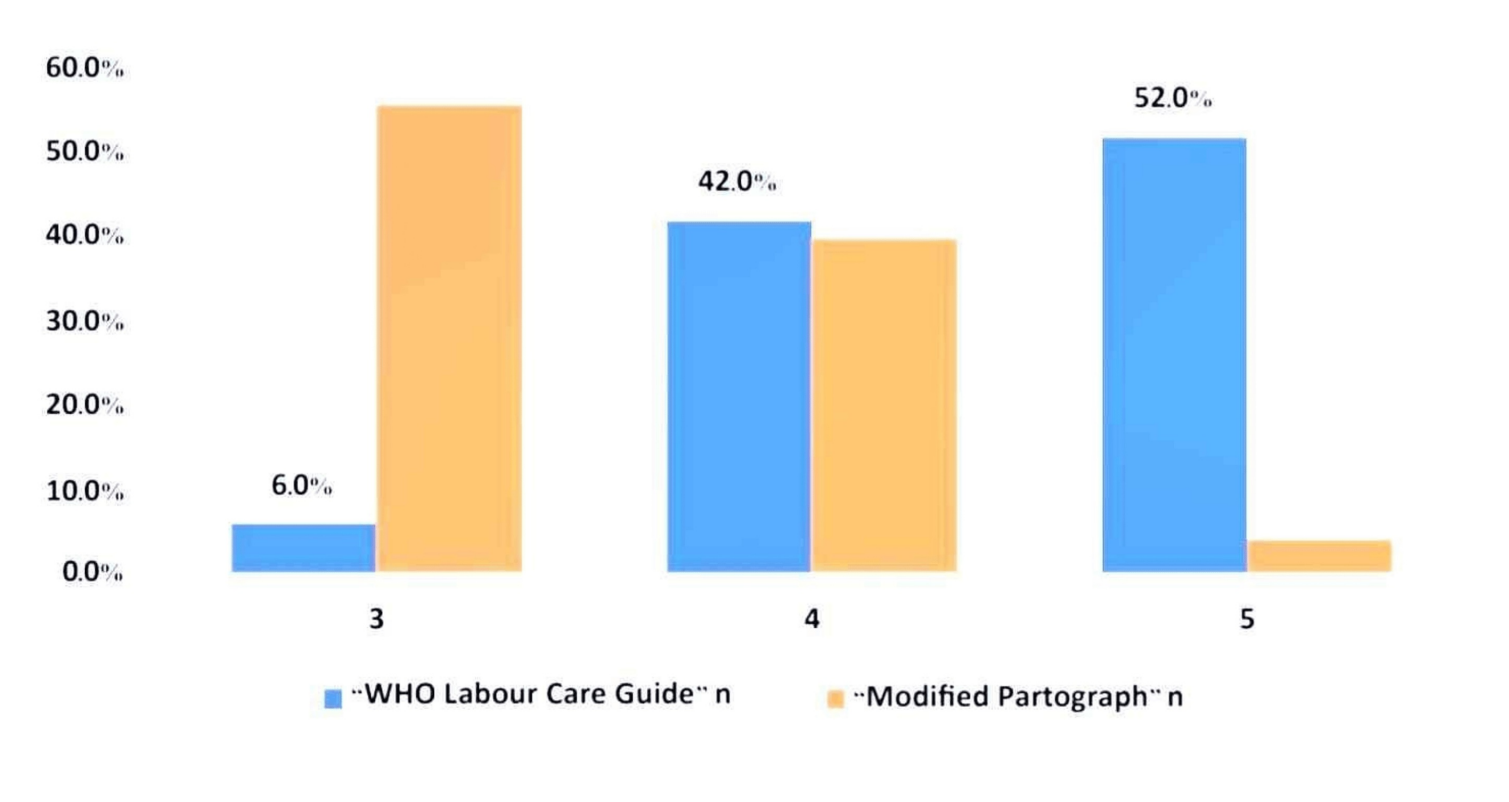
Women's experience at birth

The difference in women’s experience at birth between the two groups was statistically significant (p = 0.000), indicating a notably more positive birth experience in the WLCG group compared to the Modified Partograph group. The Likert scale was used to assess women's experience at birth. No patients reported experience ratings of 1 or 2 on the Likert scale (Figure 3). Women's birth experience ratings were significantly better in the WLCG group (p < 0.001).

**Figure 4:**
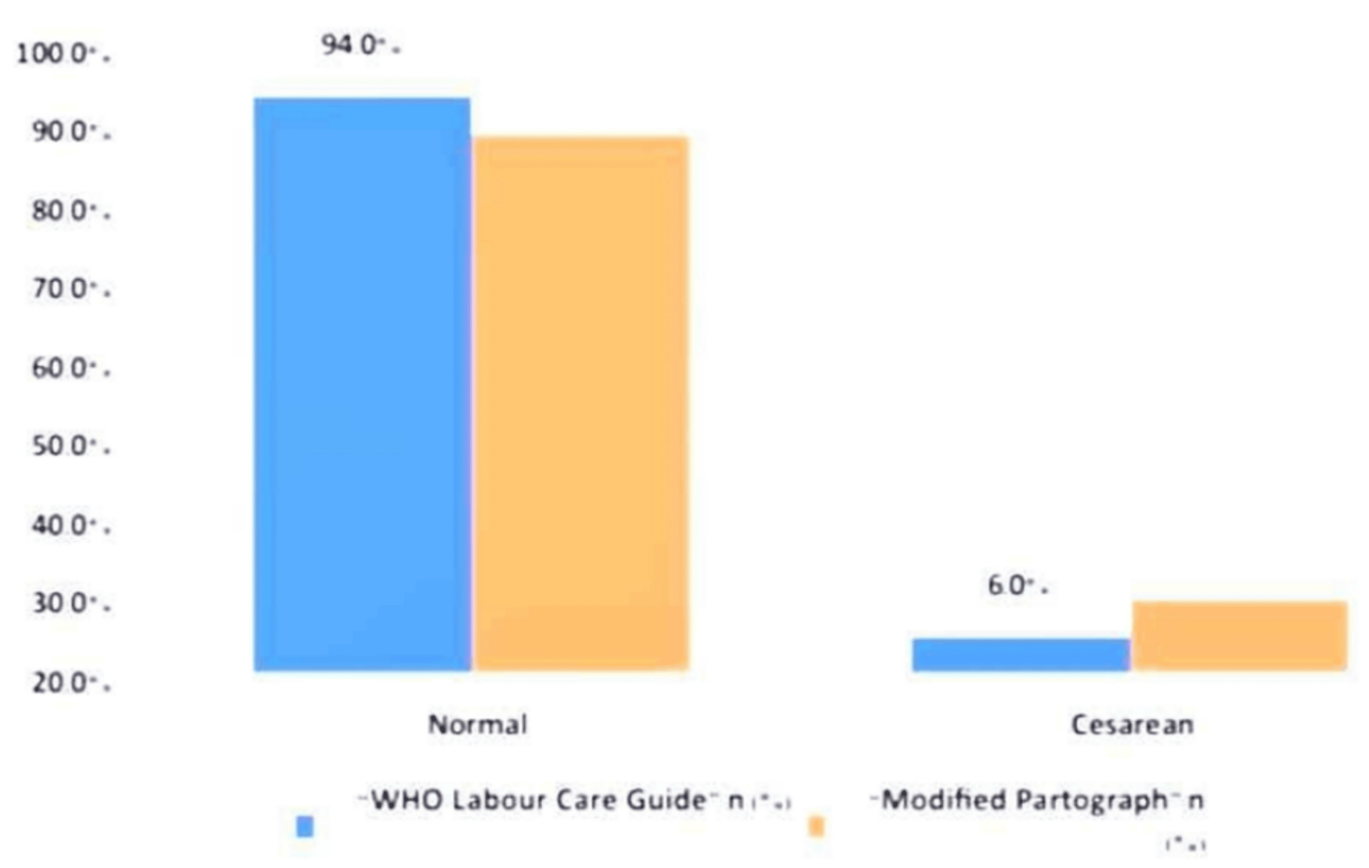
Mode of delivery in each group

Normal vaginal delivery rates were slightly higher in the WLCG group (94%) compared to the Modified Partograph group (88%), though not statistically significant (p = 0.295) (Figure 4).

Causes for labour intervention (foetal distress and non-progress) were similar between groups (p = 0.743). Foetal outcomes showed no significant difference in APGAR scores (p = 0.907) or neonatal mortality (p = 0.603). However, NICU admissions were significantly lower in the WLCG group (30% vs. 52%, p = 0.025) (Tables 5-7). Maternal complications, including postpartum haemorrhage (PPH) and sepsis, showed no significant difference (p = 0.419), indicating a comparable incidence of maternal complications in both groups. 

**Table 5:** Requirement of NICU admission in each group

Requirement of NICU Admission	WHO Labour Care Guide, n (%)	Modified Partograph, n (%)	p-value
Yes	15 (30.0%)	26 (52.0%)	0.025
No	35 (70.0%)	24 (48.0%)	
Total	50 (100%)	50 (100%)	

**Table 6:** Foetal outcome in each group

Foetal Outcome	WHO Labour Care Guide, n (%)	Modified Partograph, n (%)	p-value
Live	48 (96.0%)	47 (94.0%)	0.603
Neonatal Death	2 (4.0%)	2 (4.0%)	
Still Birth	0 (0.0%)	1 (2.0%)	
Total	50 (100%)	50 (100%)	

**Table 7:** APGAR score in each group

Group	APGAR Score Mean ± SD
WHO Labour Care Guide	7.70 ± 0.84
Modified Partograph	7.68 ± 0.87
Total	7.69 ± 0.85
p-value	0.907

## Discussion

This study highlights the advantages of using the WLCG over the Modified Partograph in rural settings. The significant reduction in the second stage of labour and improved women's birth experiences suggest that the WLCG promotes better physiological labour progression and more respectful maternity care. These findings support those of Hofmeyr et al. [5], who questioned the traditional one-centimetre-per-hour rule used in the “Modified Partograph,” proposing a more individualised approach similar to that of the LCG. The lower NICU admission rates in the WLCG group point towards its effectiveness in improving immediate neonatal outcomes. This finding is similar to studies showing the poor documentation and completion rates associated with partographs [6], which may contribute to delays in identifying neonates at risk.

Our findings are consistent with WHO recommendations promoting woman-centred care and active monitoring to reduce unnecessary interventions and enhance maternal satisfaction. The comparable rates of maternal complications and neonatal mortality between the groups confirm the safety of both monitoring tools. This supports previous research [7], which indicated that the LCG fosters woman-centred care and is highly accepted by practitioners.

The mode of delivery results indicate that the LCG and the “Modified Partograph” have comparable effectiveness in facilitating normal vaginal deliveries (94.0% vs. 88.0%, p = 0.295). This finding is similar to Vogel et al.'s (2021) study [7], in which 91.6% of women under LCG monitoring had a spontaneous vaginal birth. Although our study showed a slightly lower rate of caesarean sections in the LCG group (6.0% vs. 12.0%), the difference was not statistically significant, suggesting that both tools provide effective labour monitoring without significantly altering caesarean section rates. However, studies such as Vogel et al. (2024) [8] suggest that the LCG might be associated with a reduction in caesarean section rates - an effect not observed in our study. 

Maternal complications were similar between groups (p = 0.419), with PPH being the most common complication in both groups. This finding is consistent with Wakgari et al. (2015) [9], who reported poor adherence to partograph documentation, which may affect the early detection of maternal complications.

Limitations of the study include variability in implementation, which encompasses training and familiarity, where health workers may be more familiar with the Modified Partograph, leading to inconsistent use or incomplete documentation when switching to the LCG. The LCG is newer and more comprehensive, requiring more training, which may affect adherence and accuracy. Study design limitations include that many studies comparing the two tools have limited sample sizes, which affects statistical power and generalisability. It is also difficult to blind providers to the tool they are using, introducing potential observer bias.

The LCG includes patient-centred care elements, like pain management and emotional support, which are harder to quantify consistently compared to the objective parameters of the Modified Partograph. Inconsistent charting or missing data points can skew comparisons. It may be better suited for settings with sufficient staff and resources, whereas low-resource settings may struggle to implement its more detailed protocols. Continuous monitoring (e.g., foetal heart rate and maternal vitals) is more emphasised in the LCG, requiring reliable equipment.

It emphasises quality of care and respectful maternity care, which are not primary focuses of the Modified Partograph. Comparing the two tools on purely clinical outcomes may overlook the patient-experience benefits. Non-inferiority or superiority: many studies aim to show that the LCG is non-inferior rather than superior, which can limit conclusions about clear advantages.

## Conclusions

The WLCG offers several advantages over the Modified Partograph, including shorter second-stage labour, enhanced maternal birth experiences, and reduced NICU admissions, without increasing maternal or foetal risks. Adoption of the WLCG in rural maternity centres is likely to improve the quality of labour management and outcomes.
